# First Order Reversal Curve Study of SmFe_2_ Melt-Spun Ribbons

**DOI:** 10.3390/ma11101804

**Published:** 2018-09-22

**Authors:** María C. Grijalva-Castillo, Carlos R. Santillán-Rodríguez, Renee J. Sáenz-Hernández, María E. Botello-Zubíate, José A. Matutes-Aquino

**Affiliations:** 1 CONACYT-Centro de Investigación en Materiales Avanzados, S.C., Miguel de Cervantes 120, Complejo Industrial Chihuahua, Chihuahua 31136, Mexico; 2 Centro de Investigación en Materiales Avanzados, S.C., Miguel de Cervantes 120, Complejo Industrial Chihuahua, Chihuahua 31136, Mexico; carlos.santillan@cimav.edu.mx (C.R.S.-R.); joselin.saenz@cimav.edu.mx (R.J.S.-H.); eugenia.botello@cimav.edu.mx (M.E.B.-Z.); jose.matutes@cimav.edu.mx (J.A.M.-A.)

**Keywords:** FORC distribution, magnetic interactions, melt spinning, intermetallic alloys

## Abstract

First-order reversal curves (FORC) and the FORC distribution provide a detailed characterization of the relative proportions of reversible and irreversible components of the magnetization of a material, revealing the dominant interactions in the system. Alloys with the nominal composition SmFe_2_ were obtained by melt-spinning with a cooper wheel velocity of 30 m/s. X-ray powder diffraction analysis showed a greater part consisting of an amorphous phase and a very small amount of SmFe_2_ crystalline phase with an average crystallite size of 8 nm. A constant acceleration Mössbauer spectrum, measured at room temperature in transmission mode, was fitted to a continuous distribution of effective fields at the nucleus of the amorphous phase (about 84% of the total area), plus two sextets for the non-equivalent sites of Fe in the SmFe_2_ crystalline phase. 91 first-order reversal curves were collected in a Quantum Design PPMS-VSM with reversal fields from –800 mT to +800 mT and using a calibration field of 850 mT. The obtained FORC diagrams showed a combined effect of a local interaction field and a mean interaction field, and showed that the reversible magnetization is a function of both, the applied magnetic field and the irreversible magnetization.

## 1. Introduction

Rare earth-Fe_2_ alloys are of great interest due to their high magnetostriction even at low magnetic fields [1]. Among them is SmFe_2_ has been reported to give the highest known negative magnetostriction of about −2000 ppm [2].

Magnetic hysteresis phenomena are often investigated by means of the classical Preisach model and more refined Preisach-type models. However, these models can be used only when certain conditions are fulfilled, for example the classical Preisach model can only be used for static hysteresis nonlinearities which exhibit the wiping-out property and congruency of minor loops [3]. In the past years, there has been an increasing interest in the application of the so-called first order reversal curves (FORC) for the analysis of magnetic hysteresis [4,5]; this approach is not model-based and the system under study is described by the FORC distribution which is directly obtained from first order reversal curves measurements. A FORC distribution provides a detailed characterization of the hysteretic response of a magnetic system to an applied field by revealing the dominant interactions in the system, the magnetic viscosity effects and the destruction of memory during a demagnetization process [6]. If the hysteresis system is modeled as consisting of microscopic switching units, each with a given coercivity, B_c_, and interaction field, B_b_, the FORC diagram corresponds to the distribution of these microscopic bias/coercivity fields [7].

A FORC is a path inside the major hysteresis loop and is obtained by taking a sample to its positive saturation state; the field is then decreased to a lower value known as reversal field, B_r_, and then is increased in small steps while measuring the magnetization, M, for an applied field, B_a_. The magnetization at the applied field B_a_ on the FORC with reversal field B_r_ is denoted by M(B_a_, B_r_), where B_a_ ≥ B_r_.

The FORC distribution is defined as the mixed second derivative:

(1) $$\;\rho \left(B_{r}{,\;B}_{a}\right)\;=\;\frac{1}{2}\frac{\partial^{2}M\left(B_{r}{,\;B}_{a}\right)}{\partial B_{r}\partial B_{a}}\;$$

The ½ factor in Equation (1) has been added to equate FORC distributions to Preisach distributions for hysterons [8,9]. To plot the FORC distribution in the B_c_ vs. B_b_ space, the following transformation equations are used [10]:

(2) $$\;B_{c}\;=\;\frac{1}{2}{(B}_{a}{+B}_{r})\;$$

(3) $$\;B_{b}\;=\;\frac{1}{2}{(B}_{a}-B_{r})\;$$

Even though a lot of information is included in the FORC diagrams, it is necessary to use an appropriate model for their correct interpretation. A model where the FORC distribution, ρ(B_c_, B_b_), is equal to the Preisach distribution in its moving variation model [11,12,13] was used for the interpretation of the SmFe_2_ FORC diagrams. This does not imply that the Preisach model describes the behavior of the interactions present in our study sample, but it is going to be used as a comparative foundation.

## 2. Materials and Methods 

Alloys with the nominal composition SmFe_2_ were prepared by arc-melting pure Sm (99.9%) and Fe (99.95%) under argon atmosphere and subsequent melt-spinning with a cooper wheel velocity of 30 m/s. An additional 3 wt % samarium was added to compensate losses by evaporation during melting. 

The composition of the melt-spun ribbons was verified by energy dispersive X-Ray spectroscopy (EDS) microanalysis performed in a JEOL JSM-7401F field emission scanning electron microscope with a magnification of 300× and a voltage of 15 kV.

The formation of crystalline phases was investigated by means of X-ray powder diffraction using a PANalytical (X’pert PRO) X-ray diffractrometer employing Cu Kα radiation in the X’Celerator detection mode. The step used was 0.17° and the time per step was 100 s. Dark field transmission electron microscopy was carried on a Philips CM200 microscope with a maximum voltage of 200 kV and electron diffraction patterns were recorded. 

Ribbons were crushed into a fine powder and a constant acceleration Mössbauer spectrum was measured at room temperature in transmission mode, using a Co^57^ source in a rhodium matrix. Mössbauer analysis was carried out with Moss Winn 3.0 software [14]. 

Magnetic properties were investigated for a single SmFe_2_ ribbon (7 mm long, 3 mm height, and 40 mm width). Hysteresis loops were measured on a single ribbon, at room temperature with a LDJ 9600 vibrating sample magnetometer (VSM); the maximum applied magnetic field was 1.5 T. The magnetic field was applied along 3 different directions of the ribbon: (i) along the largest side of the plane, (ii) along the shortest side of the plane and (iii) normal to the plane (see insert in Figure 5). The demagnetizing factor was calculated using the method proposed by Chen, et al. [15].

**Figure 1:**
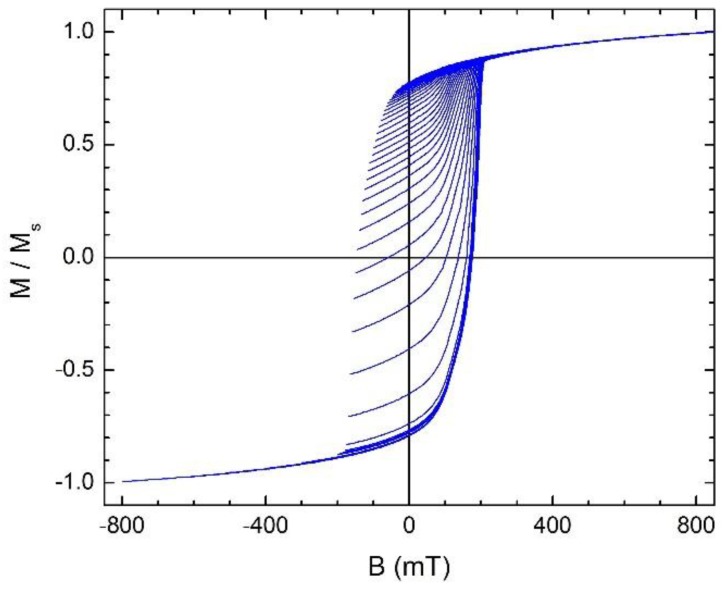
A set of measured first order reversal curves plotted in normalized magnetization vs. applied magnetic field.

A suite of 91 first-order reversal curves was collected in a Quantum Design Physical Properties Measurement System with a VSM probe, using the procedure described in Section 1. The magnetic field was applied in plane along the easy axis of the sample, the saturation field was 850 mT and the reversal field range was from 800 mT to −800 mT with a sweep rate of 5 mT/s. To avoid the interference of the magnetic viscosity effects with the FORC measurements, an interval of 4 min was awaited at each reversal field, B_r_, before measuring each FORC. All measured FORCs are shown in Figure 1.

The FORC distribution, diagrams and curves were obtained with the FORCIT Software [16]. The datasets generated during and/or analyzed during the current study are available from the corresponding author on reasonable request.

## 3. Results and Discussion

### 3.1. Microstructure

The composition of the melt spun ribbons was verified by EDS, showing to be close to that of the SmFe_2_ intermetallic alloy (Table 1). 

**Figure 2:**
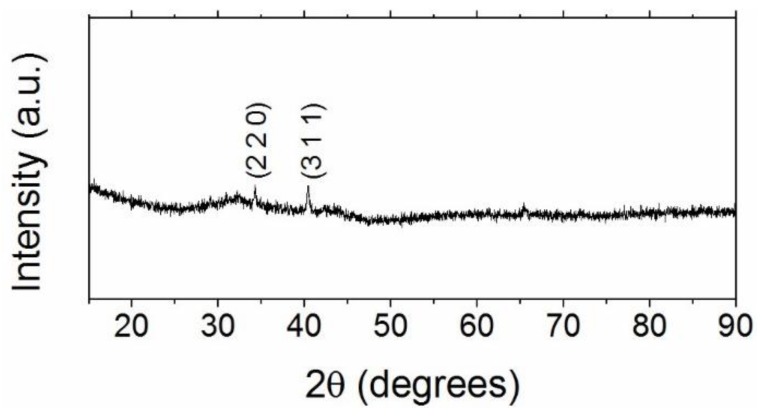
X-ray powder diffraction of SmFe_2_ melt-spun ribbons.

X-ray powder diffraction analysis (Figure 2) showed a greater part consisting of an amorphous phase and small peaks at 34.2° and 40.33°. These reflections were indexed to the SmFe_2_ crystal structure, space group Fd3m (227), lattice parameter a = 7.415 Å.

**Figure 3:**
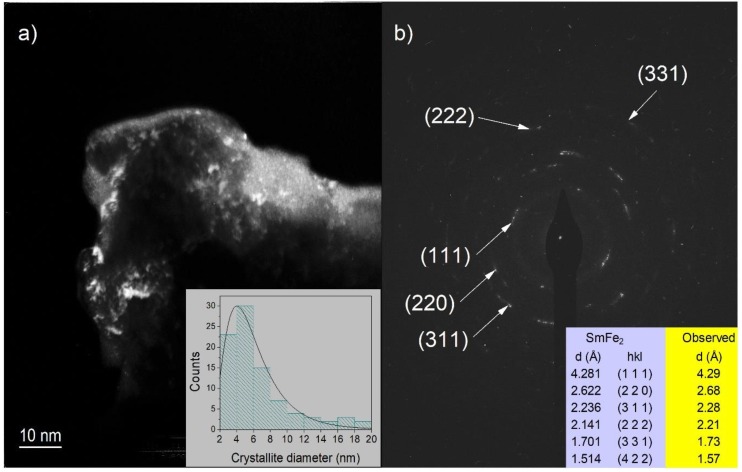
(**a**) Dark field TEM micrography of the obtained melt spun ribbons. (**b**) Diffraction pattern of the observed crystalline zones in the sample. The diffractions of intermetallic SmFe_2_ are shown for comparison purposes.

Dark field transmission electron microscopy (Figure 3a) indicated that the samples consist of some crystals surrounded by an amorphous matrix. The statistical analysis of the crystalline zones (insert in Figure 3a) showed that the crystallite size is smaller than 20 nm, the average crystallite size is around 6 nm and most of the crystals have sizes below 8 nm.

Diffraction patterns of the crystalline zones (Figure 3b) were indexed to diffractions of the intermetallic compound SmFe_2_, International Centre for Diffraction Data, PDF-2, entry #025-1152 [17]. 

**Figure 4:**
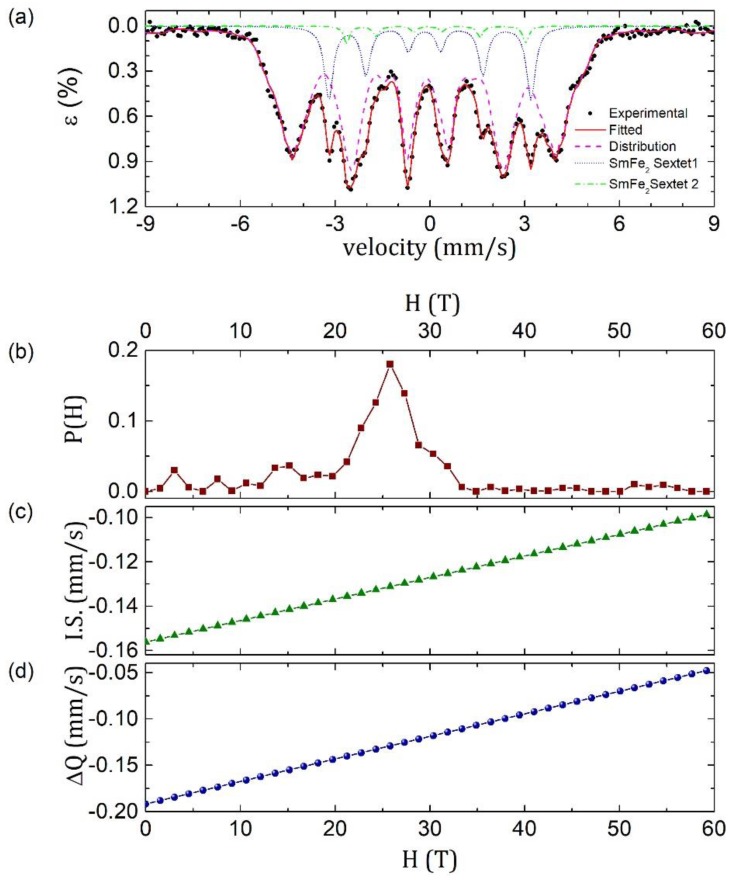
(**a**) Mössbauer spectrum collected and fitted to an amorphous phase and to SmFe_2_ phase, (**b**) distribution function of effective magnetic fields for the amorphous phase, (**c**) isomer shift linear function for the amorphous phase, and (**d**) quadrupolar splitting linear function for the amorphous phase.

The obtained Mössbauer spectrum (Figure 4a) was fitted to a continuous distribution of effective magnetic field at the Fe^57^ nucleus of the amorphous phase, plus two sextets corresponding to the two non-equivalent crystallographic sites of Fe in the SmFe_2_ Laves phase [18]. The area analysis showed that 84 wt % of the sample correspond to the amorphous phase, and 16 wt % to the SmFe_2_ Laves phase.

For the crystalline portion of the sample, a model where an axially symmetric electric field gradient tensor with symmetry axis parallel to the magnetic field was assumed [19]. In this model, the spin is defined I = 3/2, and the energy, *E*, is:

(4) $$\;E\;=\;-g\mu_{n}Hm_{I}+{\left(-1\right)}^{\left|m_{I}\right|+\frac{1}{2}}eqQ/4\;$$

where $g\mu_{n}H$ is the splitting between adjacent levels, *m_I_* is the magnetic quantum number and ¼ eqQ is the even displacement of all four magnetic sublevels due to the quadrupolar interaction. The calculated hyperfine parameters for the crystalline phase and for the continuous distribution of effective magnetic fields are shown in Table 2.

### 3.2. Magnetic Characterization

**Figure 5:**
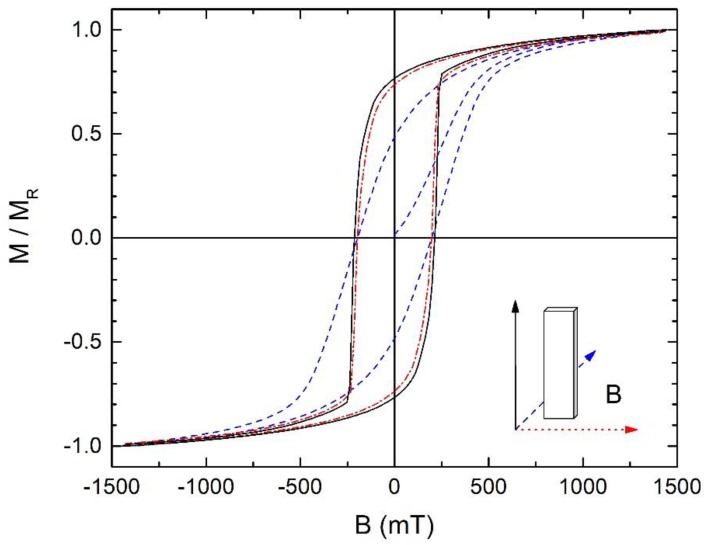
Hysteresis loops measured at room temperature along the longitudinal, transversal and perpendicular relative directions of magnetic field with the ribbon.

Hysteresis loops were measured applying the magnetic field in the three different relative directions with the ribbon: longitudinal (the longer side of the plane), transversal (the shorter size of the plane) and perpendicular with the plane of the ribbon (Figure 5).

The measured magnetization, at the field of 1.5 T, was 36.6 Am^2^/kg for the longitudinal direction, 26.5 Am^2^/kg for the transversal direction, and 26.3 Am^2^/kg for the perpendicular direction. This change in saturation magnetization is an indication that a preferred easy magnetization axis orientation is created during melt spinning along the longitudinal direction of the ribbons. Additionally, an abrupt magnetization reversal is observed at the applied magnetic field around the coercivity value, about 215 mT. 

**Figure 6:**
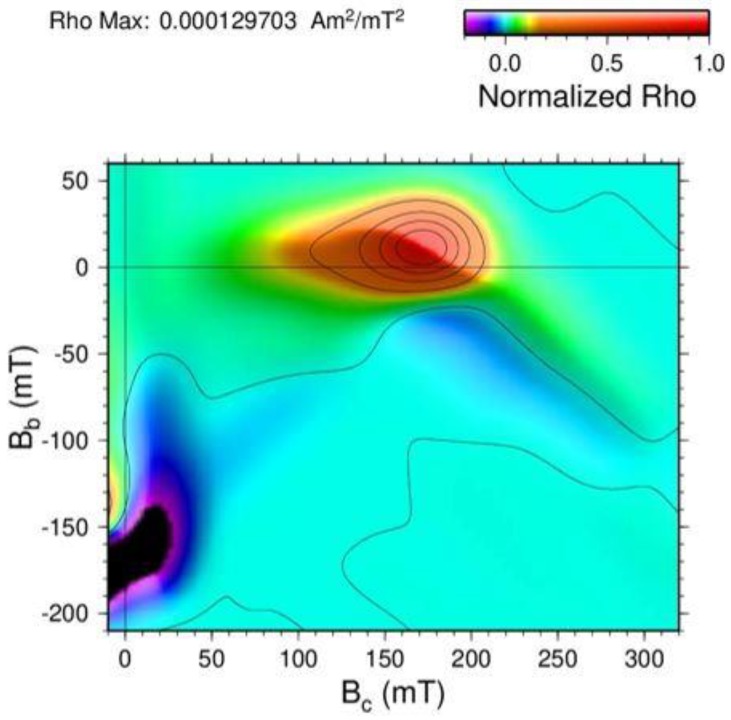
FORC diagram of SmFe_2_ ribbon. The Rho distribution is normalized to ρmax = 0.000129703 Am^2^/mT^2^.

From the set of measured FORC’s (Figure 1), the Rho distribution was calculated (Equation (1)) and the FORC diagrams obtained (Figure 6). The coordinate axes of the FORC diagrams are expressed in coercivity field (B_c_) and interaction field (B_b_). The shape of the Rho distribution gives information about the different kinds of interactions present in the sample. The main peak on the Rho distribution, centered approximately at B_c_ = 172 mT and B_b_ = 10 mT, has contributions from the SmFe_2_ nanocrystals and from the amorphous region that surround them. 

The classic Preisach model [3] assumes the system as formed by non-interacting, single-domain particles, and with this model the FORC distribution has a main peak centered in (B_c_, B_b_ = 0) with no broadening of the peak in the interactions axis, B_b_. However, the introduction of a randomly changing interaction field, as in the moving Preisach model [11], causes a broadening of the main peak along the interactions axis B_b_. This can be seen as the vertical spread of the FORC distribution in Figure 6, along the interactions axis. 

If the action of a mean interaction field, kM, is taking into account, a displacement of the main peak from B_b_ = 0 is produced and there is also a positive or negative slope in its contour. This displacement occurs toward positive values of B_b_ when k is negative and vice versa [12]. As this effect does not contribute to the broadening of the main peak, it is possible to separate the effects of the local interaction field from those of the mean interaction field. In our case of study, the displacement of the center of the distribution above B_c_ = 0 and its negative slope are both indications of a negative mean interaction field kM. 

The “wing-like” area and the negative area of the Rho distribution around B_c_ = 200 mT and B_b_ = −50 mT was explained by Pike, et al. [13] as the action of: (1) a collection of symmetric hysterons with a distribution of coercivities, and (2) a simple antiparallel mean interaction field. As stated before, melt-spun ribbons have a magnetic texture along the longest side of the ribbon. This will create interactions of demagnetizing nature along the SmFe_2_ nanocrystals, whereas positive interactions of a magnetizing nature are more dominant within the sample due to the majoritarian amorphous phase. 

**Figure 7:**
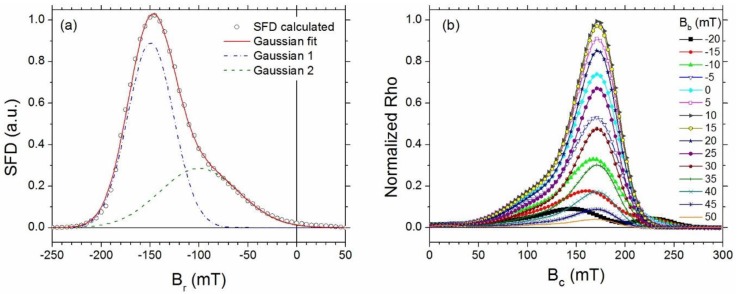
(**a**) Projection of the switching field distribution (SFD) onto the Br axis obtained by integrating over Ba. (**b**) Rho distribution plots for constant values of interaction field, B_b_, from −20 mT to +50 mT.

The switching field distribution (SFD) can give insight to the onset and endpoints of irreversibility [20]. The SFD is the projection of the FORC distribution ρ(B_r_, B_a_) onto the B_r_ axis (Figure 7a). Such a projection is equivalent to integrating ρ along B_a_, leading to:

(5) $$\;\int \frac{\partial^{2}{M(B}_{r}{,B}_{a})}{\partial B_{r}\partial B_{a}}{\mathrm{dB}}_{a}\;=\;\frac{{\mathrm{dM}(B}_{r})}{{\mathrm{dB}}_{r}}\;$$

The SFD was fitted to a pair of Gaussian functions (continuous line in Figure 7a) and the area associated to each function was calculated. The first Gaussian function (about 63% of the total area) is associated with the amorphous phase that is not exchange coupled. The second Gaussian function (about 37% of the total area) is associated with the exchange coupled nanocrystals and with those nanocrystal been exchanged coupled with the amorphous phase surrounding them. Since the average ratio of the nanocrystals is in the order of the exchange length, we can assume that the total volume of nanocrystals is exchanged, coupled with the amorphous phase. 

Several plots of the main peak of the FORC distribution for constant values of interaction field B_b_ from −20 mT to 50 mT are shown in Figure 7b. As it can be observed the maximum value of the Rho distribution corresponds to an interaction field B_b_ = 10 mT, where the coercivity B_c_ is around 172 mT. This value is very close to that of the measured bulk coercivity of 175 mT. 

The different values of B_b_ in the Rho distribution are associated with the wide range of local atomic environments within the amorphous matrix that are not exchange coupled with the nanocrystals. However, a considerable reduction in the interaction fields can be noticed and is related to the fraction of amorphous phase that is exchange coupled with the nanocrystals and is due to the more defined atomic environments in the nanocrystalline phase.

**Figure 8:**
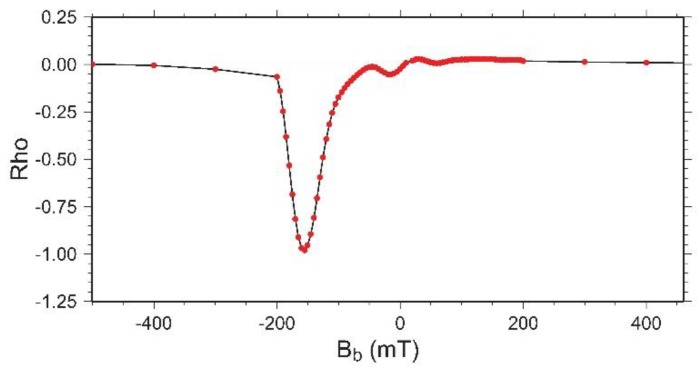
Reversible ridge for Bc = 0.

The Rho distribution along a profile where B_c_ = 0 (Figure 8) is known as reversible ridge because is due to the presence of reversible magnetization. This reversible ridge is associated with the rotation of low-coercivity particles [21]. 

One can observe that the reversible ridge is not symmetric about B_b_ = 0. This lack of symmetry is due to the reversible magnetization being coupled not only to the applied field, but also to the irreversible state of the system. On the other hand, the negative values of Rho in the left bottom region (darker color) of Figure 6 are also associated with the non-symmetric shape of the reversible ridge about B_b_ = 0.

## 4. Conclusions

First order reversal curves were used for the analysis of magnetic hysteresis in SmFe_2_ melt spun ribbons, revealing the dominant interactions in the systems. The main characteristics of the FORC distribution are: (i) the irreversibility region with a slightly negative slope and its center shifted to positive values of interaction field, (ii) the spread in the interaction field distribution, which decreases with lower values of coercivity, (iii) the reversibility ridge along the B_b_ axis (B_c_ = 0) with a non-symmetrical FORC distribution about B_b_ = 0, (iv) the region with negative values of the FORC distribution near B_b_ = −70 mT, B_c_ = 10 mT. These features can be explained by the combined effect of a local interaction field, a mean interaction field kM with negative k, and a reversible magnetization as a function of the applied magnetic field and also as function of the irreversible magnetization. 

The contribution of the SmFe_2_ nanocrystals and the contribution of the amorphous region that surround them can be split from the SFD showing that the totality of the nanocrystals are exchange coupled with a fraction of the amorphous phase that surround them and 37% of the total volume of the sample is exchange coupled. From the FORC distribution analysis can be deduced that positive interactions of magnetizing nature are more dominant within the sample due to the majoritarian amorphous phase, but also the demagnetizing effect of the small fraction of nanocrystals can be noticed.

## Figures and Tables

**Table 1:** EDS of the ribbon sample measured in different regions.

	Nominal SmFe_2_	Sample Region 1	Sample Region 2	Sample Region 3	Sample Region 4
at% Sm	33.3	34.18	32.68	33.82	34.62
at% Fe	66.6	65.82	67.32	66.18	65.38

**Table 2:** Hyperfine parameters of the SmFe_2_ sample.

	Magnetic Field (T)	Isomer Shift (mm s^−1^)	Quadrupolar Splitting (mm s^−1^)	Line Width (mm s^−1^)	Amplitude
SmFe_2_ sextet 1	19.8	−0.089	0.172	0.33	33576.8
SmFe_2_ sextet 2	17.6	0.065	0.261	0.20	8286.6
Distribution	24.4	−0.130	−0.132	0.35	75837.3
		m = 0.00097 b = −0.1562	m = 0.00243 b = −0.1917		

## Supplementary Files

Supplementary File 1
