# Supplementary material for: First Order Reversal Curve Study of SmFe2 Melt-Spun Ribbons
Source: Materials (Basel). 2018 Sep 22;11(10):1804. doi: 10.3390/ma11101804 (PMC6212980; doi:10.3390/ma11101804)
Supplement: Supplementary File 1 [file materials-11-01804-s001.pdf]

Cristina Grijalva

Author: administrator
Creation: 9/13/2017

Sample Name: SN05

Added Spectra

9/13/2017 10:59:59 AM

kV: 15	Mag: 300	Takeoff: 34.8	Live Time(s): 48.3	Amp Time(µs): 7.68	Resolution:(eV)130.4

9/13/2017 10:59:59 AM

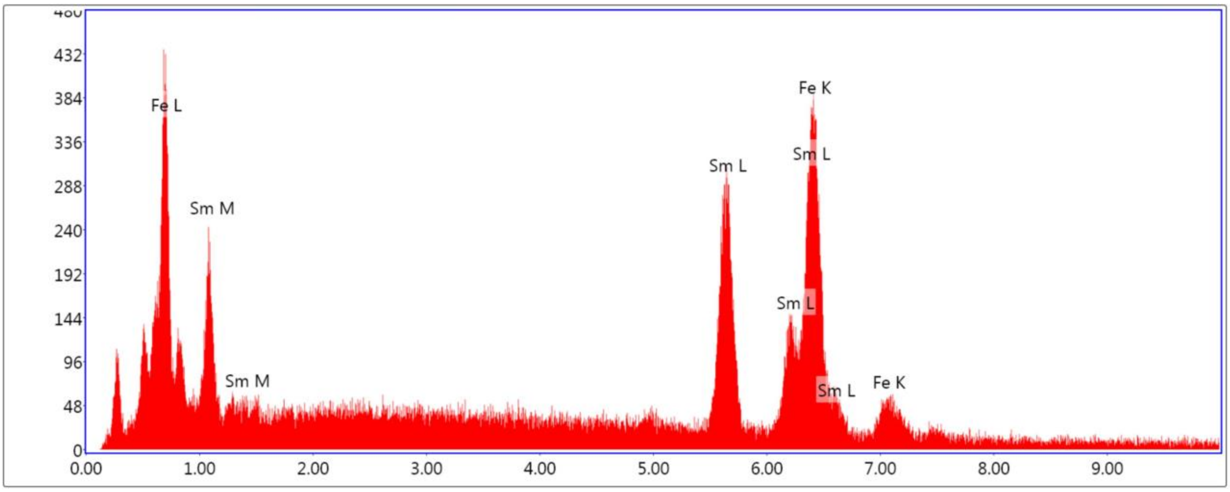

Element	Weight %	Atomic %	Net Int.	Error %
SmL	58.3	34.18	156.02	7.97
FeK	41.7	65.82	206.7	4.66

Cristina Grijalva

Author: administrator
Creation: 9/13/2017

Sample Name: SN05

Added Spectra

9/13/2017 11:06:40 AM

kV: 15	Mag: 300	Takeoff: 34.8	Live Time(s): 48.2	Amp Time(µs): 7.68	Resolution:(eV)130.4

9/13/2017 11:06:40 AM

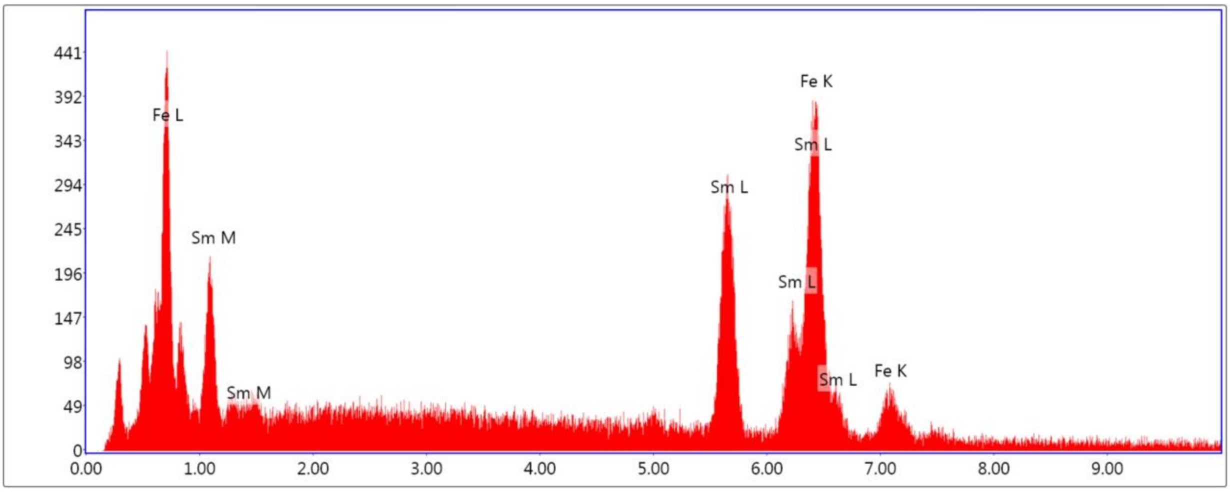

Lsec: 48.2 0 Cnts 0.000 keV Det: Octane Super Det

Element	Weight %	Atomic %	Net Int.	Error %
SmL	56.65	32.68	153.11	8.03
FeK	43.35	67.32	216.8	4.65

Cristina Grijalva

Author: administrator
Creation: 9/13/2017

Sample Name: SN05

Added Spectra

9/13/2017 11:09:48 AM

kV: 15	Mag: 300	Takeoff: 34.8	Live Time(s): 48.3	Amp Time(µs): 7.68	Resolution:(eV)130.4

9/13/2017 11:09:48 AM

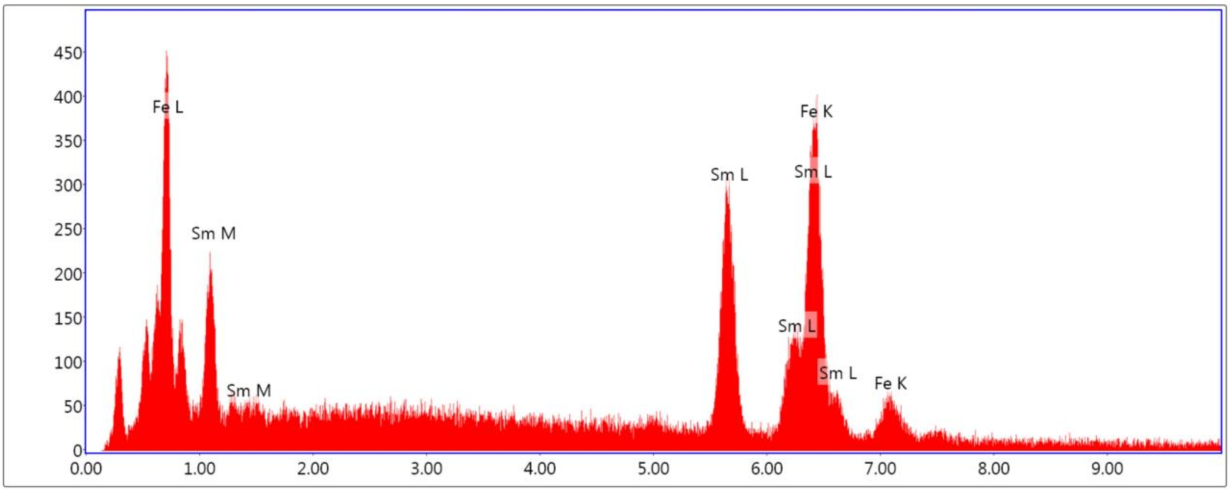

Lsec: 48.3 0 Cnts 0.000 keV Det: Octane Super Det

Element	Weight %	Atomic %	Net Int.	Error %
SmL	57.91	33.82	156.84	8.08
FeK	42.09	66.18	211.13	4.59

Cristina Grijalva

Author: administrator
Creation: 9/13/2017

Sample Name: SN05

Added Spectra

9/13/2017 11:12:57 AM

kV: 15	Mag: 300	Takeoff: 34.8	Live Time(s): 48.1	Amp Time(µs): 7.68	Resolution:(eV)130.4

9/13/2017 11:12:57 AM

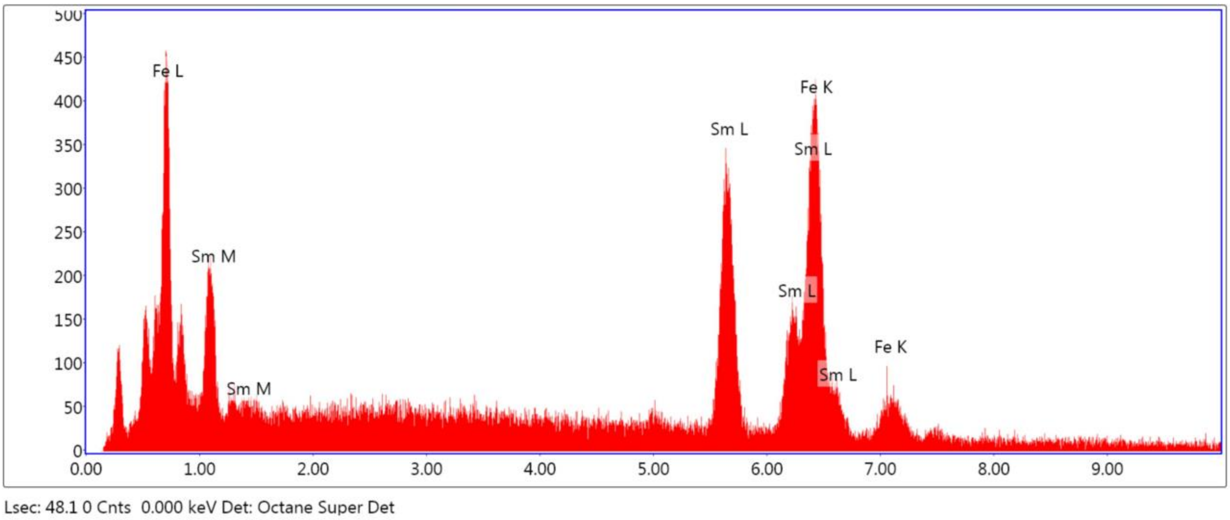

Element	Weight %	Atomic %	Net Int.	Error %
SmL	58.77	34.62	175.68	7.25
FeK	41.23	65.38	228.4	4.54
